# Barriers to Childhood Immunisation in Selected Zero-Dose Communities in Gauteng Province: A Qualitative Study

**DOI:** 10.3390/vaccines14050439

**Published:** 2026-05-14

**Authors:** Thobelani Nompilo Majola, Ntombifuthi Blose, Emma Shuvai Chikovore, Zinhle Mtwane, Algernon Africa, James Michael Burnett, Maanda Mudau, Noluthando Ndlovu, Bontle Motloung, Janine Simon-Meyer, Ashnie Padarath

**Affiliations:** 1 Research and Implementation Science, Health Systems Trust, Durban 3630, South Africa; ntombifuthi.blose@hst.org.za (N.B.); chikovoree@gmail.com (E.S.C.); zinhle.mtwane@hst.org.za (Z.M.); algernon.africa@hst.org.za (A.A.); michael.burnett@hst.org.za (J.M.B.); maanda.mudau@hst.org.za (M.M.); noluthando.ndlovu@hst.org.za (N.N.); ashnie.padarath@hst.org.za (A.P.); 2 Health and Nutrition Section, United Nations International Children’s Emergency Fund (UNICEF) South Africa, Pretoria 0011, South Africa; bmotloung@unicef.org (B.M.); jasimon@unicef.org (J.S.-M.)

**Keywords:** barriers, childhood immunisation, zero-dose, urban, metropolitan area, Gauteng, South Africa

## Abstract

**Background/objective**: The Immunisation Agenda 2030, led by the World Health Organization, aims to ensure that people of all ages benefit from vaccination. South Africa remains committed to these goals, strengthening childhood immunisation largely through the Expanded Programme on Immunisation. However, despite progress, the number of unvaccinated and partially vaccinated children continues to rise in some urban settings. This study sought to identify barriers to childhood immunisation in selected zero-dose urban communities in Gauteng Province. **Methods**: A qualitative exploratory–descriptive design was used to examine factors influencing childhood immunisation. Data were collected through seven focus group discussions and fifteen key informant interviews with purposively selected caregivers, community leaders, community health workers and healthcare workers involved in routine immunisation services at public healthcare facilities across the Cities of Johannesburg, Ekurhuleni, and Tshwane. Thematic analysis was conducted using NVivo^®^ software. **Results**: The study identified multiple demand-side and supply-side barriers. Demand-side barriers included limited parental knowledge, lack of trust in the government and immunisation services, religious beliefs, migration, and perceptions of caregiver substance use. Supply-side barriers involved distance to the facility, negative healthcare worker attitudes, long queues and waiting times, and frequent vaccine stock-outs. These barriers collectively contributed to missed opportunities and decreased uptake of immunisation services. **Conclusions**: Childhood immunisation remains a critical and cost-effective public health intervention. The findings demonstrate the complex interplay of individual and system-level factors influencing vaccine demand, uptake and persistent zero-dose status in urban Gauteng communities. Addressing these barriers requires a comprehensive approach that strengthens community trust, improves caregiver vaccine literacy, and ensures accessible, responsive, and respectful immunisation services.

## 1. Introduction

Child immunisation is one of the most cost-effective public health interventions for the reduction of child morbidity and mortality [1]. The Immunisation Agenda 2030 (IA2030) is a progressive global initiative endorsed by all 194 World Health Organization Member States, recognising immunisation as a universal human right, essential for achieving physical and mental health, investing in future generations, and creating a healthier environment for all [2]. IA2030 guides immunisation efforts across countries of all income levels, including settings such as South Africa, India, Brazil, and Nigeria, where reducing inequities in vaccine coverage remains a key priority. Strategic priority 3 of IA2030 specifically emphasises equitable access to immunisation for all children and communities in order to reduce morbidity and mortality from vaccine-preventable diseases (VPDs) [3,4]. South Africa launched the Expanded Programme on Immunisation (EPI-SA) and introduced the Hepatitis B vaccine in 1995, other vaccines, targeting various infectious diseases, were progressively introduced over the 30-year period [5,6]. The overarching goal of EPI-SA is to provide quality services to ensure that all children within targeted age groups receive potent vaccinations and are protected from illness [6,7].

The impact of South Africa’s immunisation programme has been considerable. In its 30 years of implementation, the programme has improved child vaccination coverage and provided a strong foundation for introducing new vaccines [7]. Routine childhood immunisation has strengthened child health outcomes by reducing illness and deaths from VPDs such as tuberculosis, polio, rotavirus, and measles. More recent national successes include catch-up vaccination campaigns, strengthened measles outbreak response activities, and continued recovery of routine immunisation services following disruptions caused by the coronavirus disease 2019 (COVID-19) pandemic [8,9,10].

Despite the significant progress in child health improvement attributed to EPI-SA, there are persistent challenges, including suboptimal immunisation coverage [11]. Estimates suggest that there were 270,602 zero-dose children in South Africa in 2024 [12]. Zero-dose children are defined as those who lack access to, or are never reached by, routine immunisation services [13]. Operationally, they are commonly identified as children who did not receive the first dose of the Diphtheria, Tetanus, and acellular Pertussis (DTaP) vaccine scheduled at six weeks of age [13]. Children may fail to access immunisation services due to multiple demand- and supply-side barriers, including limited caregiver awareness, vaccine hesitancy, financial and transport constraints, vaccine stockouts, long waiting times, and poor provider–client interactions [14,15].

These immunisation gaps have had measurable public health consequences. In the first half of 2025, the Gauteng Department of Health reported 181-laboratory confirmed measles cases, many originating from Cities of Johannesburg, Tshwane and Ekurhuleni. This outbreak followed previous clusters dating back to 2022 and was largely fuelled by zero-dose and partially immunised children in high-density districts and metropolitan areas, highlighting the systemic consequences of this “immunity gap” [5]. Similarly, in January 2024 to March 2025, a situation report by the National Institute of Communicable Diseases (NICD) reported 31 cases of diphtheria; of these cases, six were among children under 12, and of those, three had vaccination history where one was partially immunised (<3 infant doses), and the others were unvaccinated for DTaP [16].

The COVID-19 pandemic exacerbated existing weaknesses, resulting in a regression of coverage rates that has left thousands of children susceptible to VPDs [17]. According to the District Health Barometer, in 2021/22, South Africa recorded the lowest under-1-year immunisation and measles second-dose coverage at 79.5% and 76.4%, respectively, attributed to the restrictions implemented during the COVID-19 pandemic [18]. While coverage improved in 2023/24, with 83.3% under-1-year coverage and 84.9% for measles second-dose coverage, both figures remain below the 90% target required for the successful elimination of childhood diseases [11,18].

Aggregate national figures, however, do not reflect sub-national disparities. Sub-national data indicate that the absolute number of zero-dose children is highest in metropolitan municipalities. Of the eight districts with the highest number of zero-dose children, five are metropolitan municipalities [19]. The persistence of large unimmunised populations within these well-resourced areas suggests that physical proximity to services does not guarantee uptake. Despite this, there is a dearth of localised and context-specific evidence investigating the drivers of low immunisation uptake. The 2024/25 District Health Barometer data further highlight variation in under-1 immunisation coverage across the study areas, with the City of Ekurhuleni at 84.2%, the City of Tshwane at 72.8%, and the City of Johannesburg at 93.0% [20]. Notably, the City of Johannesburg has achieved the IA2030 target of 90% coverage, while Cities of Ekurhuleni and Tshwane remain below this benchmark.

Existing South African studies have largely focused on rural districts, national trends, or quantitative coverage estimates, with less attention given to the lived experiences of caregivers and healthcare workers in urban zero-dose settings. The purpose of this study was to explore and describe the perspectives of healthcare workers, caregivers, and community members on barriers to childhood immunisation access in the metropolitan municipalities of Ekurhuleni, Johannesburg, and Tshwane. While demand- and supply-side barriers are well-documented globally [1,15,21,22,23], limited evidence exists on how these determinants specifically converge to drive the rising zero-dose rates within these complex, high-density urban environments.

## 2. Materials and Methods

### 2.1. Study Setting

The study was carried out in three metropolitan municipalities in South Africa, namely the Cities of Johannesburg, Ekurhuleni, and Tshwane, all located in Gauteng Province, and all having high proportions of zero-dose children. The three municipalities were among the top eight districts with the highest number of zero-dose children and, collectively, constituted approximately 16% (*n* = 44,604) of the national total [19].

The City of Johannesburg is the most populous metropolitan municipality and the country’s largest economic hub [24]. As of 2022, the City of Johannesburg was reported to be home to 4,803,262 people, of whom those aged 0–4 years composed 8.7%, representing the primary population requiring routine immunisation services [25]. It is also one of the most rapidly growing and urbanised cities in the country and in Africa [26]. In 2024/25, an estimated 22,832 children in the City of Johannesburg were classified as zero-dose, representing approximately 5.5% of the estimated population of children aged 0–4 years [27].

The City of Ekurhuleni Metropolitan Municipality in Gauteng Province has a population of 4,066,691 people. Males constitute 51% of the population, while children aged 0–4 years make up 8.4% [25]. The city is highly urbanised, with 99.4% of the population living in urban settings ranging from informal settlements to elite urban residential suburbs [28]. In 2024/25, an estimated 15,964 children were zero-dose in the City of Ekurhuleni, representing approximately 4.7% of the estimated population of children aged 0–4 years [27].

The City of Tshwane Metropolitan Municipality in northern Gauteng is the province’s largest by size. It had a population of 4,040,315 in 2022, up from 3,275,152 in 2016, with males comprising 50.1% [25,29]. The municipality has 1,322,252 households, 13.1% being informal dwellings, averaging 3.1 members [25]. Most of the city’s population is between 15 and 64 years old (70.9%), children between 0 and 4 years old constitute the second largest population group (23.0%), and older people 65+ years constitute 6.1% of the population [25]. In 2024/25, an estimated 11,607 children were zero-dose in the City of Tshwane, representing approximately 1.25% of all children [27].

A total of three facilities were selected as research sites in each metro. The selection of facilities was based on a consultation with the Gauteng Department of Health, including district and operational managers. During these consultations, subdistricts and facilities were identified based on routine immunisation data and local programme knowledge, particularly in relation to communities with a high proportion of zero-dose children. Facilities located in urban areas with documented immunisation coverage gaps and service delivery challenges were prioritised to ensure alignment with the study’s focus on urban zero-dose populations.

### 2.2. Study Design

The study employed a qualitative exploratory–descriptive design to gain an in-depth understanding of the opinions, perspectives, and experiences of caregivers and healthcare workers regarding childhood immunisation uptake [30]. This design was used to identify and describe factors contributing to low immunisation uptake and zero-dose status in selected urban communities. A qualitative exploratory–descriptive approach is appropriate for examining issues that are insufficiently understood or where limited empirical evidence exists, as it enables the collection of rich, context-specific accounts from participants and facilitates deeper insight into complex social and health system phenomena [31]. This design was therefore suitable for the present study, as barriers affecting immunisation uptake in zero-dose and underserved urban settings in Gauteng remain underexplored despite their significant implications for child health and public health programming.

### 2.3. Data Collection

Data were collected through seven focus group discussions (FGDs) and 15 key informant interviews (KIIs) conducted between 10 and 28 March 2025 across selected public primary healthcare facilities in the metropolitan municipalities of Johannesburg, Ekurhuleni, and Tshwane. Semi-structured interview and discussion guides were used to collect qualitative data on participants’ knowledge, perceptions, experiences, and barriers related to childhood immunisation uptake (Supplementary File S1).

Purposive sampling was used to recruit participants with relevant experiences and perspectives regarding childhood immunisation. Eligible participants were adults aged 18 years and older who were caregivers of a child under one year of age, healthcare workers; community leaders; faith leaders; traditional health practitioners; community health workers; andrepresentatives of migrant and other community groups. Recruitment was undertaken in consultation with healthcare workers at the selected facilities, who assisted in identifying and inviting eligible participants.

FGDs were conducted with caregivers, including young parents and grandparents, and each group consisted of 8–10 participants. Three FGDs were conducted in the City of Johannesburg, while two FGDs each were conducted in the City of Ekurhuleni and City of Tshwane. KIIs were conducted with key stakeholders, with five interviews undertaken in each metropolitan municipality.

All KIIs and FGDs were facilitated by trained fieldworkers in private spaces within healthcare facilities after obtaining informed consent. Interviews were conducted in English, audio-recorded, and transcribed for analysis.

### 2.4. Data Analysis and Trustworthiness

Qualitative data were systematically collected and transcribed, with quality assurance procedures implemented to ensure data integrity. The analysis was conducted using NVivo^®^ version 14 software (Lumivero, Burlington, VT, USA). The qualitative data analysis software was used to apply thematic analysis in order to identify salient patterns within the dataset. An inductive approach was employed, whereby the data were systematically reviewed to identify emerging codes, patterns, and themes. The analysis was guided by Braun and Clarke’s six-step framework for thematic analysis, which includes familiarisation with the data, coding, generating themes, reviewing themes, defining and naming themes, and producing the final report [32]. The analytic process focused on barriers to childhood immunisation as reported during KIIs and FGDs. Emergent themes were organised into two principal categories: demand-side and supply-side barriers.

It is important to ensure trustworthiness in all qualitative studies. Various strategies were applied to address credibility, transferability, dependability, and confirmability. Credibility was achieved through persistent observation of the collected data, allowing the researcher to understand the complex experiences of caregivers and healthcare providers [33]. This process ensured that data was assessed without bias and interpretations remained accurate. Transferability was supported by providing thick descriptions of the urban contexts, socio-economic conditions, and participant characteristics. Dependability was maintained through an audit trail documenting all methodological decisions and peer debriefing sessions to review processes and interpretations. Finally, confirmability was ensured through reflexive journaling, in which researchers documented their assumptions, decisions, and reflections throughout the study. Raw data and coding notes were also preserved for external audit, thereby minimising bias and enhancing objectivity.

### 2.5. Ethical Considerations

The study was reviewed and approved by Pharma-Ethics, a registered Independent Ethics Committee (Reference Number 241126736), and permission to conduct the study was obtained from the Gauteng Provincial Health Research Ethics Committee. Additionally, district support letters were obtained from each metropolitan municipality. Participants in the KIIs and FGDs provided written informed consent prior to their participation. The informed consent process was completed with all participants, who were informed about the purpose of the study, their rights, and the voluntary nature of participation. This included consent for audio recording. Only the project team had access to personally identifiable information, which was securely stored and used solely for study administration. During data capturing and analysis, participants were allocated unique codes, and all data were analysed collectively to protect individual identities. Audio recordings and notes were handled confidentially, and all study data will be stored securely for five years and permanently deleted thereafter. All participants were informed about the recordings, note-taking, and the expectation that discussions remain confidential. All participants were also given an opportunity to ask questions or raise any concerns before participating.

## 3. Results

### 3.1. Sociodemographic Characteristics of Study Participants

Table 1 presents the demographic characteristics of KII and FGD participants. Seventy-eight participants were recruited for the study, including 15 for KIIs and 63 for seven FGDs with 8 to 10 participants each. Participants were drawn from selected facilities across three metropolitan municipalities: 39.7% (*n* = 31/78) from the City of Johannesburg, 32.1% (*n* = 25/78) from the City of Tshwane, and 28.2% (*n* = 22/78) from the City of Ekurhuleni. The most frequently reported age group among participants in the City of Johannesburg was 36–45 years old (25.8%; *n* = 8/31), while in the Cities of Tshwane and Ekurhuleni, the most frequently reported group was young parents aged 18–25 years (40%; *n* = 10/25 and 40.9%; *n* = 9/22, respectively). Most participants in all metropolitan municipalities were female (93.6%; *n* = 73/78). Only five participants were male, including one from the City of Johannesburg and two each from the City of Ekurhuleni and the City of Tshwane, respectively. The majority of the participants in all metropolitan municipalities had a high school education (67.9%; *n* = 53/78). Eighty-eight percent (*n* = 22/25) of participants in the City of Tshwane had a high school education compared to the Cities of Johannesburg and Ekurhuleni, which had 71% (*n* = 22/31) and 41% (*n* = 9/22) of participants respectively. In terms of employment status, 66.7% (*n* = 52/78) of participants reported that they were unemployed, while 33.3% (*n* = 26/78) reported being employed.

### 3.2. Themes

Multiple barriers emerged from the study findings. These were categorised into demand-side and supply-side barriers (see Table 2).

#### 3.2.1. Demand-Side Barriers

The analysis highlighted five prominent demand-side barriers to childhood immunisation. These are parental lack of knowledge, lack of trust in vaccine safety, the influence of religious beliefs, migration, and the impact of substance abuse.

##### Lack of Parental Knowledge

Parental access to information emerged as a key factor shaping attitudes towards childhood immunisation. In several instances, limited understanding of vaccines and immunisation schedules acted as a barrier to service uptake. Parents and caregivers in the metros reported uncertainty or insufficient knowledge about the purpose, safety, and benefits of childhood vaccines, which contributed to discomfort and hesitancy in seeking immunisation services for their children.

*“They don’t give us lessons or information about childhood vaccination; we cannot immunise children without information.”*

(Grandparent, City of Ekurhuleni, FGD)

*“We just know that there are vaccines, but we are not sure why they are important for children, the difference between a child who is immunised and the ones who are not.”*

(Young parent, City of Johannesburg, KII)

Some parents and caregivers also demonstrated limited knowledge of common vaccine side-effects. When children experienced reactions such as swelling at the injection site, some parents interpreted this as evidence that vaccines were harmful or that healthcare workers had administered them incorrectly. Additionally, when parents asked healthcare workers about vaccine contents and some were unable to provide clear explanations, this undermined confidence in both the vaccines and the providers. As a result, uncertainty about side-effects and perceptions of inadequate healthcare workers’ knowledge contributed to poor parental knowledge, trust and further reduced parents’ willingness to immunise their children.

*“When my child was vaccinated for the first time, she got swollen, more like she was bitten by a spider. When I went to the clinic, they could not answer what was the cause and what was in the vaccine, how can I trust vaccines if health workers also don’t have answers.”*

(Young parent, City of Johannesburg, FGD)

*“Some parents lack knowledge about vaccines, which is why they do not vaccinate children. We have witnessed parents not willing to vaccine children.”*

(Healthcare worker, City of Johannesburg, KII)

##### Perceived Lack of Trust in Vaccine Safety and the Government

Parents and caregivers expressed mixed perceptions regarding childhood vaccines. While some viewed the vaccines as safe and necessary, others questioned their safety and associated them with adverse health outcomes, such as skin rashes. Concerns were also raised about perceived insufficient government transparency regarding vaccine components and potential side-effects, which contributed to declining trust and reduced vaccine uptake. In addition, some parents and caregivers doubted the competence of healthcare workers, particularly those involved in community-based vaccination activities.

*“My second one didn’t go for vaccination, and she was okay until six months and then after she started vaccinating. When she started the vaccine, she started having rash and skin problem, I think it is because of the vaccine.”*

(Young parent, City of Johannesburg, FGD)

*“The sisters or nurses conducting school-based vaccination programmes, are they trained? I am asking because there was a child who was infected with measles after he got vaccinated at the school.”*

(Grandparent, City of Ekurhuleni, FGD)

Trust in vaccine safety and efficacy was often undermined by scepticism rooted in misinformation and past COVID-19 vaccination experiences, particularly among older adults. Confusion between routine childhood immunisation and COVID-19 vaccines further complicated acceptance. Trust emerged as a critical factor influencing decisions about vaccine uptake. Limited knowledge about childhood vaccines and the distinctions between different types of vaccines also undermined trust and contributed to hesitancy. Some parents and caregivers believed that routine childhood immunisation was linked to COVID-19 vaccines, leading to reluctance to vaccinate their children.

*“Since there were COVID-19 vaccines that did not treat people well, we are just not comfortable to vaccinate children. There are many speculations about vaccines.”*

(Faith leader, City of Ekurhuleni, KII)

*“The elderly have doubts since the COVID-19 vaccines were introduced. They’re confusing child vaccination with COVID-19 vaccinations, they are reluctant to take their grandchildren to clinics for vaccines.”*

(Community leader, City of Tshwane, KII)

##### Religious Beliefs

Findings showed that decisions not to vaccinate children were shaped by religious beliefs. Certain religious teachings discouraged vaccine uptake and undermined trust by prohibiting vaccination and expressing disapproval of vaccines.

*“They don’t mention immunisation in church; they don’t support it.”*

(Young parent, City of Ekurhuleni, FGD)

*“It’s just that in other churches it is something that is not discussed, some do not encourage it, and this makes people not to trust vaccines.”*

(Young parent, City of Johannesburg, KII)

The Islamic Medical Association of South Africa promotes childhood immunisation and affirms that vaccination aligns with Islamic principles of preserving life and preventing harm. Despite this institutional support, concerns rooted in religious beliefs were reported by some Muslim participants and contributed to vaccine hesitancy. Several participants expressed fears that vaccines may contain pork-derived or otherwise impermissible (haram) ingredients, which they believed conflicted with their religious practices. In some instances, faith-based objections resulted in complete avoidance of vaccination, highlighting the strong influence that personal and community-level religious interpretations can have on healthcare decisions.

*“Some members of the local Indian community didn’t come because they don’t want immunisation. They say it contains pork.”*

(Healthcare worker, City of Johannesburg, KII)

*“There’s some medication that is made from pork like maybe they’ll take a scrap of the skin or something, that’s why the Muslim community doesn’t want measles vaccine because it is against our belief.”*

(Muslim community caregiver, City of Tshwane, KII)

##### Migration

The findings indicate that language barriers and experiences of mistreatment were significant factors hindering immunisation uptake among migrant populations. Migrant participants reported that discrimination within healthcare settings undermined their trust in health services, leading to reluctance to seek immunisation for their children. One parent described a situation in which healthcare workers refused to assist her because of a language barrier, insisting she return with a translator for future visits. This experience affected her confidence in the health system and made her sceptical about continuing with her child’s vaccinations. Another participant echoed these concerns, saying that she felt migrants were often treated differently compared to other patients.

*“Nurses told me that if you don’t speak my language, we are not going to help you. This demotivated me because I could not speak their language. I am now hesitant to immunise my child because I don’t trust their services.”*

(Young parent, City of Tshwane, FGD)

*“I feel like they treat foreigners differently than the locals, that’s not good because at the end of the day, they are here for services and should be treated well and equally.”*

(Muslim community caregiver, City of Ekurhuleni, KII)

Migrants’ attitudes were also perceived to be a barrier influencing immunisation demand. Participants said some migrant families refused to vaccinate their children due to religious and cultural beliefs rooted in their countries of origin.

*“Some migrants do not believe in vaccines due to their beliefs, how they were brought up and where they come from.”*

(Grandparent, City of Ekurhuleni, FGD)

*“We sometime come across migrants who are not willing to immunise children even when we explain the importance of vaccines.”*

(Healthcare worker, City of Johannesburg, KII)

##### Perceptions of Substance Use

Issues related to substance use were also highlighted, particularly in the City of Ekurhuleni. Community health workers reported that during home visits, some parents and caregivers who had missed vaccination appointments were perceived to be using substances. They described this as contributing to inconsistent caregiving, lower prioritisation of children’s health needs, and difficulties maintaining regular immunisation schedules.

*“When we conduct home visits, we often come across parents who have missed vaccination appointments and most of them are substance users.”*

(Community health worker, City of Ekurhuleni, KII)

*“Issues around substance abuse among some parents affect vaccination uptake, we observe this in communities.”*

(Healthcare worker, City of Ekurhuleni, KII)

#### 3.2.2. Supply-Side Barriers

Supply-side barriers to childhood immunisation include health system factors that affect the availability and quality of services, such as long distances to health facilities, poor quality of care, negative healthcare worker attitudes, and vaccine stock-outs.

##### Distance to the Facility

Access to healthcare services was widely reported as a barrier to immunisation uptake. Parents and caregivers described multiple challenges, including long distances to clinics, high transport costs, and poor communication from facilities. These difficulties often led to delayed immunisation visits, with many preferring outreach services to avoid travel-related expenses. For some caregivers, commuting to health facilities was especially burdensome, as they were unable to afford taxi fares, further limiting timely access to childhood vaccines.

*“Sometimes we don’t have money for transport, and this is something that is really needed. Sometimes we miss appointments because of money.”*

(Grandparent, City of Ekurhuleni, FGD)

*“I’m a bit far and there’s no clinic closeby. When I don’t have money, I miss some immunisation appointments.”*

(Young parent, City of Tshwane, KII)

##### Quality of Care

The quality of care experienced at health facilities played an important role in shaping parents’ and caregivers’ decisions to immunise their children. Several factors contributed to dissatisfaction with services, including unclear process flows within facilities, long waiting times, and the absence of queue marshals to guide and support patients. These challenges collectively created frustration and discouraged some caregivers from returning for immunisation services.

*“I wasn’t happy with the service, the clinic is always full and the lines are long. We queue for long hours before getting vaccines and this is tiring.”*

(Traditional health practitioner, City of Ekurhuleni, KII)

*“The long queues were discouraging, I would end up hungry because of the long waiting times.”*

(Young parent, City of Johannesburg, FGD)

##### Negative Healthcare Worker Attitude

Interactions with healthcare workers played a central role in shaping parents’ and caregivers’ experiences at health facilities, as well as their trust in and use of immunisation services. However, negative attitudes from some healthcare workers undermined this trust, particularly when staff failed to explain the potential side-effects of childhood vaccines. In several instances, caregivers perceived this lack of communication as indifference or a lack of concern for their children’s well-being. Such experiences contributed to a reluctance to return for follow-up doses and weakened overall confidence in the immunisation programme.

*“When I had a child, I was a teenager, they would always make comments which discouraged me to take my child for immunisation.”*

(Young parent, City of Tshwane, KII)

*“Days are not the same, sometimes you get lucky to find the sister who is patient but sometimes you get someone who is so impatient.”*

(Grandparent, City of Ekurhuleni, FGD)

##### Vaccine Stock-Out

Vaccine stock-outs posed significant challenges to service delivery across multiple healthcare facilities. Respondents indicated that such shortages often resulted in considerable discouragement among parents and caregivers, who frequently travelled long distances, incurred transportation costs, or took time off work, only to find that the required vaccines were unavailable. These stock-outs not only resulted in wasted time and financial strain but also eroded confidence in the reliability of immunisation services. For some caregivers, repeated experiences of stock-outs reduced their motivation to return for subsequent visits, thereby contributing to missed or delayed vaccinations.

*“Sometimes the immunisation is not even there, you travel using your last cent to sit in long queues and the child ends up not receiving the vaccine, it is demoralising.”*

(Young parent, City of Tshwane, FGD)

*“We travel long distances and take leave from work only to find that vaccines are not available. We cannot rely on these facilities.”*

(Faith leader, City of Ekurhuleni, KII)

## 4. Discussion

### 4.1. Demand-Side Barriers

Barriers influencing demand for immunisation are diverse, yet each has the potential to disrupt vaccine acceptance and uptake. When left unaddressed, these barriers may contribute to rising numbers of zero-dose and partially immunised children. This study identified several challenges that hinder childhood immunisation, with parental vaccine knowledge and beliefs emerging as consistent barriers across all metropolitan municipalities. These findings align with the substantial body of literature showing that limited parental knowledge and specific parental characteristics are key determinants of childhood vaccine demand. For instance, an earlier systematic review of studies conducted in sub-Saharan Africa between January 1988 and December 2019 highlighted caregivers’ lack of knowledge about immunisation, including awareness of adverse events following immunisation as one of the most prominent barriers to vaccine uptake [34]. Similar patterns have been observed in other studies examining the factors that impede demand for, and uptake of, childhood immunisation [35,36].

Trust in vaccine safety and efficacy has long been recognised as a key determinant of vaccine demand in various contexts, as diminished confidence in vaccines often leads to hesitation or refusal [15,37,38]. When caregivers question the safety or effectiveness of vaccines, they are more likely to avoid immunisation altogether, reinforcing patterns of vaccine hesitancy. In the present study, concerns about vaccine safety and efficacy were identified consistently across all metropolitan cities, indicating that this is a widespread and persistent barrier to childhood vaccination. These findings align with previous research showing that parents and caregivers who harbour doubts about vaccine safety or efficacy are less likely to vaccinate their children or ensure that their children are fully immunised [39,40]. Similarly, Galagali and colleagues note that safety- and efficacy-related hesitancy continues to undermine vaccination efforts globally [41].

In addition to concerns about vaccines themselves, trust in government also emerged as an important influence on vaccine demand and uptake. Low trust in government was identified as a barrier across all metropolitan municipalities in this study. This finding echoes evidence from a study conducted in the City of Johannesburg, where lack of trust, particularly in governmental institutions, was shown to contribute to COVID-19 vaccine hesitancy [42]. Comparable results were observed in a multi-country study conducted in Botswana, the Dominican Republic, and Greece, which found notably lower levels of trust in the government among participants in Greece, demonstrating that trust-related barriers can vary widely across settings [43].

Beyond gaps in knowledge and understanding, social and cultural factors, particularly religious beliefs, also play a crucial role in shaping caregivers’ vaccination decisions. Religious beliefs emerged as an important barrier to childhood immunisation, with some caregivers declining vaccines due to mistrust of vaccine contents or perceptions rooted in longstanding religious teachings. In several cases, these beliefs were reinforced by family examples, such as unvaccinated relatives who appeared healthy. The study findings reflect evidence from other contexts, including a study conducted in Thailand among people with Muslim faith, which showed that concerns about vaccine ingredients and halal compliance contribute to hesitancy among certain religious groups [44], while family influence has also been documented as a barrier to vaccine demand in both systematic and qualitative studies [14,45].

The experiences of migrant populations also emerged as a notable barrier to childhood immunisation. Previous research has shown that migrants often face unique challenges that impede vaccine uptake. For instance, a study conducted in Sweden among undocumented migrants found that precarious migration status disrupted adherence to immunisation schedules, as caregivers avoided health facilities due to fears of arrest or deportation [46]. Similarly, evidence from Nigeria suggests that migrant communities are often disadvantaged in accessing vaccination services [47]. Findings from the current study echo these patterns. Migrants’ negative attitudes and beliefs were shaped in part by perceived mistreatment at health facilities and difficulties in communicating with healthcare workers due to language barriers, both of which discouraged the use of immunisation services. These challenges were particularly evident in the three metropolitan municipalities in Gauteng, a province characterised by high levels of in-migration.

The study findings indicate that substance use emerged as an important barrier to childhood immunisation. This finding is particularly noteworthy as it highlights how caregivers’ personal characteristics and behaviours can directly influence a child’s ability to remain up to date with vaccine schedules. Interviews revealed that some children missed routine vaccinations because their parents or caregivers were actively using substances, which affected their capacity to prioritise or consistently attend immunisation appointments. Although research directly examining the role of substance abuse in routine childhood immunisation is limited, studies focusing on COVID-19 and other forms of immunisation have consistently shown that people who use drugs have lower vaccination rates compared to the general population [48,49]. These findings underscore the need for targeted support mechanisms such as strengthened community outreach, integration of immunisation with substance-use services, and tailored counselling.

### 4.2. Supply-Side Barriers

Accessibility of healthcare services, interactions with healthcare workers, and the overall experience of care at health facilities are critical health system factors that shape vaccine demand and uptake. All three issues were consistently reported across the metropolitan municipalities included in this study, underscoring the shared challenges within these settings. Limited access, driven by long distances to facilities, transport costs, vaccine stock-outs, and prolonged waiting times, was widely identified as a barrier to immunisation. These findings align with earlier studies that have documented access-related constraints as significant impediments to vaccine uptake [15,34,50].

Another prominent health system factor highlighted in this study was the nature of interactions with healthcare workers. Negative experiences, including perceived rudeness, dismissive attitudes, and a lack of motivation among staff, discouraged parents and caregivers from bringing their children for immunisation appointments. These findings are consistent with evidence from South Africa and India, where interactions with healthcare workers were found to be pivotal in determining vaccine demand and uptake [14,37].

Furthermore, broader experiences of poor-quality care, such as disorganisation within facilities, recurring vaccine stock-outs, and generally inadequate service delivery, contributed to dissatisfaction and diminished trust in immunisation services. Similar observations have been reported in studies from Southern Ethiopia and South Africa, where negative provider–client experiences have been shown to undermine immunisation efforts by fostering reluctance toward childhood vaccination [51,52].

Collectively, these findings illustrate that barriers to childhood immunisation are multifaceted, spanning individual, social, and health system dimensions. While caregivers’ knowledge, beliefs, and lived experiences shape vaccine demand, systemic challenges within the health sector further compound these barriers. Addressing immunisation gaps, therefore, requires a comprehensive approach that strengthens health system performance, improves the quality of provider–client interactions, and fosters trust in vaccination services.

Notably, these barriers persist despite the relatively high density of healthcare facilities in urban settings, suggesting that physical proximity alone is insufficient to ensure immunisation uptake. Instead, service quality, reliability, respectful care, and trust appear to be more influential determinants of caregiver behaviour in metropolitan contexts. These finding challenges assumptions that urban residence inherently confers better access to preventive health services and underscores the need for urban-specific immunisation strategies.

It is also important to note that this study considered childhood vaccination as a general concept, which may have limited the ability to capture differences in perceptions of specific vaccines. Some participant responses, including those reflecting religious considerations, suggested that certain vaccines may be more acceptable than others, highlighting the need for future research to explore vaccine-specific perceptions.

### 4.3. Study Limitations

This study has some limitations that should be considered when interpreting the findings. First, the qualitative design and purposive sampling approach are not statistically representative of the population in the metropolitan municipalities. Second, data collection was conducted in English, which may have constrained full participation, including among some migrant caregivers. Third, participants were recruited through public health facilities, potentially excluding the perspectives of caregivers who have completely disengaged from the health system and those who have never engaged with the public health system. The study did not purposely enrol caregivers of under-vaccinated or zero-dose children; this may constitute proxy-reporting and not fully reflect the views of the most affected population. Findings may be subject to social desirability bias, which can be difficult to detect and control during qualitative research. The underrepresentation of male caregivers, particularly single fathers, may have limited the diversity of perspectives captured and reduced the comprehensiveness of findings related to caregiver decision-making. However, despite these limitations, the study provides rich, context-specific insights that are valuable for informing urban immunisation strategies.

## 5. Conclusions and Recommendations

Childhood immunisation remains a vital and cost-effective public health intervention. This study identified a range of factors influencing the uptake of immunisation services in the three selected metropolitan municipalities. These included persistent determinants such as parental knowledge, trust in health services and government, and religious beliefs, as well as emerging challenges such as substance use and the experiences of migrant populations. Together, these factors contribute to the risk of incomplete immunisation and zero-dose children, highlighting the need for targeted, context-specific interventions to improve coverage.

To address these barriers, a multi-level response is recommended across the community, facility, and health system levels. At the community level, strengthened health communication and engagement strategies are needed, including peer support networks for caregivers; involvement of community, religious and traditional leaders; and targeted messaging through local media and social media platforms to improve knowledge and counter misinformation. At the facility level, provision of user-friendly educational materials, improved patient flow systems, and ongoing in-service training for healthcare workers on respectful communication and immunisation guidelines are recommended to enhance service delivery and client experience. At the health system level, strengthening vaccine supply management, including through strategies such as the Reaching Every District (RED) approach, is essential to address persistent stock-outs and ensure consistent vaccine availability at the facility level. In addition, improving immunisation data systems (including electronic tracking and follow-up mechanisms) and enhancing coordination with private healthcare providers are essential to improve continuity, completeness, and reliability of immunisation services.

## Figures and Tables

**Table 1:** Demographic characteristics of KII and FGD participants.

	City of Johannesburg *n* = 31	City of Ekurhuleni *n* = 22	City of Tshwane *n* = 25	Total *n* = 78
Age group				
18–25	6 (19.4%)	9 (40.9%)	10 (40%)	25 (32.1%)
26–35	5 (16.1%)	2 (9.1%)	5 (20%)	12 (15.4%)
36–45	8 (25.8%)	2 (9.1%)	5 (20%)	15 (19.2%)
46–55	7 (22.6%)	3 (13.6%)	5 (20%)	15 (19.2%)
56–65	1 (3.2%)	3 (13.6%)	-	4 (5.1%)
66+	4 (12.9%)	3 (13.6%)	-	7 (9%)
Sex				
Male	1 (3.2%)	2 (9.1%)	2 (8%)	5 (6.4%)
Female	30 (96.8%)	20 (90.9%)	23 (92%)	73 (93.6%)
Marital status				
Single	22 (71%)	14 (63.6%)	14 (56%)	50 (64.1%)
Married	6 (19.3%)	6 (27.3%)	9 (36%)	21 (26.9%)
Divorced/separated /widowed	3 (9.7%)	2 (9.1%)	2 (8%)	7 (9%)
Level of education				
No/primary education	-	1 (4.5%)	-	1 (1.3%)
High school	22 (71%)	9 (41%)	22 (88%)	53 (67.9%)
Higher education	9 (29%)	12 (54.5%)	3 (12%)	24 (30.8%)
Employment status				
Employed	11 (35.5%)	2 (9.1%)	13 (52%)	26 (33.3%)
Unemployed	20 (64.5%)	20 (90.9%)	12 (48%)	52 (66.7%)
Total	31 (39.7%)	22 (28.2%)	25 (32.1%)	78 (100%)

**Table 2:** Summary of emerged themes on the barriers to childhood immunisation.

Phenomenon of Interest	Category	Themes
Barriers to childhood immunisation	Demand-side	Lack of parental knowledge
		Lack of trust in vaccine safety and government
		Religious beliefs
		Migration
		Perceptions of substance use
	Supply-side	Distance to the facility
		Negative healthcare worker attitude
		Quality of care
		Vaccine stock-out

## Data Availability

The original data are available upon request from the corresponding author.

## Supplementary Materials

The following supporting information can be downloaded at https://www.mdpi.com/article/10.3390/vaccines14050439/s1, File S1: Key Informant Interview Guide.

## Abbreviations

The following abbreviations were used in the manuscript:

COVID-19	Coronavirus disease 2019
DTaP	Diphtheria, Tetanus, and acellular Pertussis
EPI-SA	Expanded Programme on Immunisation in South Africa
FGD	Focus group discussion
IA2030	Immunisation Agenda 2030
KII	Key informant interview
NDoH	National Department of Health
NICD	National Institute of Communicable Diseases
RED	Reaching Every District
VPD	Vaccine-preventable disease
UNICEF	United Nations Children’s Fund
