# Supplementary material for: Barriers to Childhood Immunisation in Selected Zero-Dose Communities in Gauteng Province: A Qualitative Study
Source: Vaccines (Basel). 2026 May 14;14(5):439. doi: 10.3390/vaccines14050439 (PMC13211367; doi:10.3390/vaccines14050439)
Supplement: Supplementary file 1 [file vaccines-14-00439-s001.zip › vaccines-4281985-supplementary.pdf]

File S1. Key Informant Interview Guide

Opening Statement / Introduction

Good morning/afternoon and welcome to the KII. Thank you for taking the time to attend to talk about childhood immunisation. My name is [xxxx] and assisting me is [xxxxx]. We work for Health Systems Trust. We are having KIIs like this in several communities across South Africa.

You were invited because you are members one of the groups listed below for this community:

- Young/adolescent parents
- Mothers/Grandmothers
- Health promoters- team leads
- Community health workers- team leads
- Muslim community
- Community leaders
- Traditional health practitioners
- Immigrant community
- Faith leaders

You would have noticed the microphone / recorder. We audio record the KII because we do not want to miss any of your comments. People often say very helpful things in these discussions and we cannot always write fast enough to get everything that is being said. Although we will be using names in the KII, we do not use any names in our reports in order to ensure your privacy and confidentiality.

Demographic Information

Sex

A	Male	
B	Female	
C	Other (please specify):	
D	Prefer not to say	

Education

A	No education	
B	Primary School	
C	High School	
D	Higher Education	

Date of birth

Yyyy/mm/dd

Race

A	Black	
B	Coloured	
C	White	
D	Indian	
E	Other (please specify):	
F	Prefer not to say	

Relationship status

A	Single	
B	Married	
C	Divorced/ widowed	
D	Living with a partner	
E	Other (please specify):	

Occupation

A	Unemployed	
B	Occasional / Seasonal worker	
C	Self-Employed	
D	Full-time Employed	

				E	Homemaker/Stay at home parent		
				F	Other (please specify):		
Number of Living Children				Have your children been immunised?			
A	0			A	Yes (Full)		
B	1			B	Yes (Partial)		
C	2			C	No		
D	3 or more						

Main Questions

1. Has your child/children been immunised?

   Notes:
   - *For yes (full), yes (partial), or no, ask why?*
   - *Look for the individual's attitude and beliefs.*
   - *Look for parent's autonomy.*

2. What are your thoughts on vaccines and immunisations for children?

   Notes:
   - *Look for the attitude and beliefs (accurate or in-accurate).*

   Probes:
   - *"Do you think they are safe and effective?"*
   - *"Are vaccines and immunisations important?"*

3. What do you think about the government and healthcare system with regards to immunisations?

   Notes:
   - *Look for perceptions regarding the trustworthiness of government and healthcare system.*
   - *We are looking to identify the strategies used by the providers or other stakeholder in order to either encourage or dissuade immunisation.*

   Probes:
   - *"Do you believe what the government tell you regarding immunisation?"*
   - *"Do you believe what the healthcare system tell you regarding immunisation?"*

4. How would you describe your experience with regards to immunisation of children? (only for those whose children have been immunised)

   Notes:

- *Look for information pertaining to the attitudes or beliefs of the individual.*

Probes:

- *"How was the experience at the clinic?"*
- *"How do healthcare workers interact with you?"*

5. What makes you bring children for immunisation? (only for those whose children have been immunised)

Notes:

- *Look for information pertaining to the parental education and beliefs.*
- *Look for knowledge pertaining to knowledge about immunisation.*
- *Look for the factors influencing parents including women's / mother's autonomy.*

Probes:

- *"Where did you hear about immunisation?"*

6. What type of information has been provided to you regarding immunisation of children?

Notes:

- *Look for information pertaining to the following factors*
  - *perceived safety and efficacy of vaccines*
  - *parental education and beliefs*
  - *general health knowledge*
  - *healthcare worker attitudes and belief*
- *We are looking to identify the strategies used by the providers or other stakeholder in order to either encourage or dissuade immunisation.*
- *Look for information regarding follow-up visits and how parents are reminded of upcoming immunization visits.*

Probes:

- *Where did you find your information?*
- *What is the source of your information, i.e. healthcare worker, radio, newspaper, family member, friends, pastor, reverend, teacher?*

7. What other factors (not discussed under question 5, 6 and 7) plays a role in your decision to either immunise or not to immunise?

Notes:

- *Look for information pertaining to the following factors*
  - *religious beliefs*
  - *social norms*
  - *access to healthcare services*
  - *logistical challenges in reaching healthcare services*
  - *healthcare worker attitudes and beliefs*
  - *women's / mother's autonomy*

- *We are looking to identify the strategies used by the providers or other stakeholder in order to either encourage or dissuade immunisation.*

Probes:

- *Are there any religious beliefs impacting your decision to immunise children? If so, please explain (mostly applicable to Muslims)*
- *Are there any challenges relating to access to healthcare services? Please explain (applicable to all participants- including migrants).*
- *How do healthcare workers treat you/patients? Are there any challenges relating to healthcare worker's attitudes when you are visiting the facility? Please elaborate.*
-

8. Can you describe the role of the community environment (ie community, family, friends, people's opinions) in your decisions regarding the immunisation of their child/children?

Notes:

- *Look for external factors that are influencing parents, such as women's / mother's autonomy, social norms etc.*

Probes:

- *"What does the community say about immunisation?"*

9. Describe your experience with access, affordability and logistical challenges in reaching the healthcare facility (or immunisation services) in your community?

Notes:

- *Here we are looking for physical barriers such as the distance to the clinic, or the cost of transport etc.*

Probes:

- *"How long does it take to get to the clinic?"*
- *"Who looks after their other children when they have to visit the clinic?"*

10. Does religion/faith/church play a role in the immunisation of children in your community?

Notes:

- *Here we are looking for any indication that the church is either for or against immunisation.*
- *Look for women's / mother's autonomy.*

Probes:

- *"What do the churches say about immunisation?"*
- *"What do the pastors/priests say about immunisation?"*

11. Do you (or your staff/team) have sufficient support in order to effectively do your job? (only for health promoters and community health workers team leads)

Notes:

- *Here we are looking for training, job aids, workload, facility flow. We are also looking for supportive supervision.*

Probes*:*

- *“What job aids and training do you (or your team) receive in order to implement the immunisation programme?”*
- *“What training and support material is needed?”*
- *“How does immunization fit into your current workload?”*
- *“How are you supported by your superiors?”*
- *“How do you support your staff/team?”*

12. In general, how are healthcare workers treated by the community?

Notes:

- *Here we are looking for community respect, celebration of achievement.*

Probes:

- *“Do you this they are valued and respected by the community?”*

13. Do you have anything to add that we have not discussed during the KII or any of the questions before we close?

Closure

We have come to the end of our KII. I would like to thank you all for taking part.
